# Editorial: Disinformation countermeasures and artificial intelligence

**DOI:** 10.3389/frai.2025.1750972

**Published:** 2025-12-11

**Authors:** Ludmilla H. Huntsman

**Affiliations:** Cognitive Security Alliance, Alexandria, VA, United States

**Keywords:** disinformation, information integrity, AI, cognitive security (CogSec), cognitive warfare, large language models (LLMs), cognitive resilience

“Wars begin in the minds of men …,” wrote U Thant, Secretary-General of the United Nations in 1968. The insight behind this statement—that while language structures reality, conflict takes shape first through narratives, ideas, and belief systems—remains no less relevant today. Humans have studied the relationship between thought, language, and mind for at least 2,500 years. In ancient times, Plato and Aristotle looked into how words relate to mental concepts and reasoning. During the Middle Ages and Early Modern period, Descartes, Locke, Leibniz, and Kant linked mental structure, representation, and logic, laying foundations for modern theories of knowledge, computation and cognition. Over the past century, this long-standing inquiry has taken shape in a diverse range of disciplines: philosophy of mind, cognitive psychology, neuroscience, psycholinguistics, artificial intelligence, and computational cognitive modeling, among others. With the rapid advancement of large language models and a race for artificial general intelligence, these fields have converged in the strategic domain of Cognitive Security (CogSec) to address the challenges of information integrity, cognitive warfare, and malign influence. State and non-state actors alike have weaponized linguistic framing, narrative engineering, and synthetic media generation in a global contest for epistemic authority: a war for reality. CogSec seeks to protect human information processing, belief formation, and decision-making by strengthening societies' cognitive resilience against disinformation, distorted reality and coercion carried out through information ecosystems.

Why does this research topic matter? Its significance emerges from a stark reality: the stakes of synthetic disinformation—systematically coordinated, AI-powered and amplified by bad actors—are not only epistemic or political. They are human, material, and often lethal. As I write this editorial, Russian soldiers launch missiles, drones, and guided bombs on Ukrainian cities for the fourth consecutive year. Russian state media justifies these war crimes domestically through narratives rooted in persistently distorted facts, heavily manipulated language and beliefs cultivated and reinforced by long-running state-directed disinformation campaigns. The tragedy illustrates an ugly truth: biased beliefs are algorithmically engineered and deployed at national scale can precipitate genocide and crimes against humanity. Disinformation kills, carries massive human suffering, and is an imminent threat to global security. It provokes and exacerbates conflict, erodes social cohesion, undermines trust in democratic institutions, and weakens societal resilience. The *Disinformation Countermeasures and AI* topic collection illustrates that while CogSec has become a critical domain, further research is needed to devise effective strategies on how to contain malign influence in the rapidly changing world.

When we began developing this Research Topic collection, the global information environment was already showing signs of destabilization. Yet during the span of its completion, the landscape has transformed more profoundly than anticipated. The acceleration of generative AI has altered not only the scale but the texture of disinformation, with interactive agents customizing and mimicking authenticity with increasing precision. Moreover, major geopolitical actors have escalated their use of information operations as instruments of statecraft. Meanwhile, the United States responded to this rapidly evolving threat with what experts described as unilateral disarmament and even surrender. After the closure of the U.S. government's main vehicle for countering foreign disinformation (GEC), along with the U.S. Agency for Global Media and other institutions, the global information sphere became even more vulnerable to malign influence operations and asymmetrically contested. With adversaries deliberately targeting cognitive, social, and institutional fault lines, this widening imbalance underscores why new research, new alliances, and new countermeasures are indispensable.

Our Research Topic will expand your understanding of the large, interdisciplinary spectrum of the topics within the field. Deepest thanks to my co-editors George Cybenko, Alexander Makarenko, and Paul Vines for their insight, leadership, and commitment to advancing this field. We extend our gratitude to all authors from Ukraine, Germany, France, United States, United Arab Emirates, United Kingdom, Bulgaria, Greece, Italy, and Switzerland who contributed to this research topic, to the reviewers whose expertise strengthened the scholarly quality of the collection, and to the editorial staff at *Frontiers in Artificial Intelligence, Frontiers in Big Data* and *Frontiers in Political Science: Politics of Technology* for their continuous support.

The ten peer-reviewed articles trace a coherent arc from conceptual foundations to concrete technical and policy responses to disinformation. Thompson and Guillory's history of the semantic hacking project distills lessons for modern cognitive security, while Deppe and Schaal's conceptual analysis of NATO's cognitive warfare framework clarifies the strategic terrain on which manipulation campaigns unfold. Paziuk et al. decode manipulative narratives in the Russia–Ukraine conflict and Zakharchenko shows how connective strategic narratives can bolster resilience, as Pilati and Venturini provide a worldwide mapping of how AI is already used in counter-disinformation practice. Romanishyn et al. and Marushchak et al. translate these insights into policy, offering recommendations for democratic resilience and regulatory lessons from Ukraine. At the technical edge, Dyachenko et al. explore LLM services for managing social communications, Tzoumanekas et al. propose a graph neural architecture search for bot detection, and Lipianina-Honcharenko et al. introduce OLTW-TEC, an online text-ensemble method for fake-news detection. Together, these contributions converge on a clear conclusion: effective counter-disinformation demands a whole-of-society approach, in which information integrity is achieved through advanced AI methods, attribution, public-private partnerships for cognitive resilience building, and adaptive democratic governance.

The challenge before us is not merely to develop more sophisticated classifiers or improved detection algorithms. It is to create cross-sector alliances to weave technology, education, societal values, and institutional frameworks into a trustworthy ecosystem. Researchers, practitioners, policymakers, and platform designers must work together to share best practices, develop transparent evaluation standards, and build open datasets and multimodal benchmarks. The work gathered in this Research Topic underscores the complexity of this challenge while pointing to pathways for technological, cognitive, and institutional innovation.

Our hope is that this Research Topic not only offers rigorous scholarship but also serves as a foundation for collective action and a catalyst for global collaboration. In a world where the integrity of information is continually tested, strengthening our cognitive and societal resilience is not merely an academic endeavor—it is a moral and strategic imperative.

